# Fever Induces Long-Term Synaptic Enhancement and Protects Learning in an Accelerated Aging Model

**DOI:** 10.14336/AD.2025.0591

**Published:** 2026-07-21

**Authors:** Fusheng Du, Qi Wan, Oleg O Glebov

**Affiliations:** ^1^Institute of Neuroregeneration and Neurorehabilitation, Qingdao University, Qingdao 266071, Shandong, China.; ^2^Faculty of Life and Health, Shenzhen University of Advanced Technology, Shenzhen, Guangdong, China.; ^3^Department of Psychological Medicine, Institute of Psychiatry, Psychology and Neuroscience, King’s College London, De Crespigny Park, London SE5 8AF, UK.

**Keywords:** fever, hyperthermia, synaptic plasticity, aging, cognitive decline

## Abstract

The physiological impact of fever on the brain remains poorly understood. Here, we demonstrate that temperature increases by yeast injection in rats (N=9) and by whole-body hyperthermia in mice (N=7) triggers structural synaptic enhancement in the prefrontal cortex through a mechanism involving AMPA-type glutamate receptors and protein translation (N=6). Repeated pyrexia induction in juvenile rats (N=9) results in synaptic strengthening that persists into adulthood, mitigating learning deficits and synaptic loss in a D-galactose model of accelerated aging (N=9-11). Our results show how common environmental conditions may shape brain function in the long-term via synaptic plasticity, warranting further exploration of thermal treatment for cognitive protection in aging.

## INTRODUCTION

Increased body temperature represents a common consequence of infection, injury, environmental exposure, and exercise, yet its physiological consequences remain poorly understood. The brain is particularly susceptible to temperature variations, *e.g.* even moderate temperature elevation can trigger febrile seizures in children [1-3]. Mechanisms of the temperature effect on the brain remain largely obscure and in want of investigation, especially as the health burden of climate change increases [4].

The synapse represents the key locus of neuronal function, and mounting evidence implicates synaptic alterations as major consequences of febrile seizures, indicating a broad long-term effect on brain circuitry [1, 2, 5, 6]. Considering that most instances of fever and hyperthermia do not result in seizures, however, the impact of moderate temperature increase on synapses, especially in the context of development and aging, remains unclear. We therefore set out to investigate the effects of moderate fever (pyrexia) and hyperthermia on brain synapses in rats and mice.

## MATERIALS AND METHODS

### Whole body hyperthermia model

Animals (mice) were randomly allocated into whole body hyperthermia (WBH) and normal body temperature (NBT) groups. The WBH group was put into a heating box, and the temperature was increased by 0.5°C every 15 min until it reached 39°C. Temperature was maintained at 39°C for 6 h, with a rest every 2 h in which all animals were placed at room temperature for 15 min while rectal temperature was measured and 0.1 ml saline was injected intraperitoneally, before returning to the 39°C environment. Rectal temperature was measured at a final rest immediately before thermal imaging. NBT was maintained at room temperature (18-22°C), with the same procedures for injection and temperature measurement as described above. All animals were sacrificed 1 h later.

### Yeast-induced pyrexia model

Animals (rats) were divided into two groups: pyrexia (yeast) and control (saline). Yeast was dissolved in saline at 37°C to make a 20% solution. The rats in the pyrexia group received subcutaneous injection of 20% yeast solution in the posterior neck to a final concentration of 10 ml/kg, and the rats in the control group received subcutaneous injection of the same volume of saline. Rectal temperature was measured at the beginning of the injection and at 1- or 2-h intervals thereafter, thermal imaging was performed 7 h after injection, and animals were sacrificed 12 h after injection.

For juvenile pyrexia induction, 4-week old rats were subjected to yeast injections on the 1st, 4th, and 7th days of the week. The animals were sacrificed either after the last injection or at 10 weeks of age.

### Accelerated aging model

At 6 weeks, animals previously subjected to juvenile pyrexia were given D-galactose at the dose of 300 mg/kg by gavage in water between 09:00-10:00 am daily for 7 weeks until 13 weeks of age; the control group was given water. Animals were sacrificed after 14 weeks following behavioral testing.

### Novel object recognition testing

At 13 weeks of age, environmental exposure was carried out at the same time of day over a 7-day period. Animals were randomly assigned and blinded to the experimenter to avoid bias. Three days before the experiment, animals were placed in the open field box to explore freely for 10 min to adapt to the environment. Placing two identical objects symmetrically in the test box on the day of the experiment ensured that the objects were not pushed. Objects were positioned 20 cm away from the test box walls and 60 cm away from each other. Animals were placed in the experimental box with their back equidistantly from the objects. The familiarization period with the objects lasted for 5 min, during which time animals were allowed to freely explore the objects within the testing arena. For 40 min after the familiarization phase, animals were returned to their home cages to facilitate memory consolidation. This was followed by a 10-min long test session, during which one of the familiar objects was replaced with a novel one and the animals' behavior was recorded on video.

Recorded videos were used to calculate the proportion of time that the animals spent exploring the old objects (F) and novel objects (N). Recognition index (RI) was calculated as RI = N/(N+F), and discrimination Index was calculated as DI = (N-F)/(N+F). Difference in recognition bouts was calculated as the number of exploration bouts for novel objects divided by the sum of the above for novel and old objects.

## RESULTS

**Figure 1.**
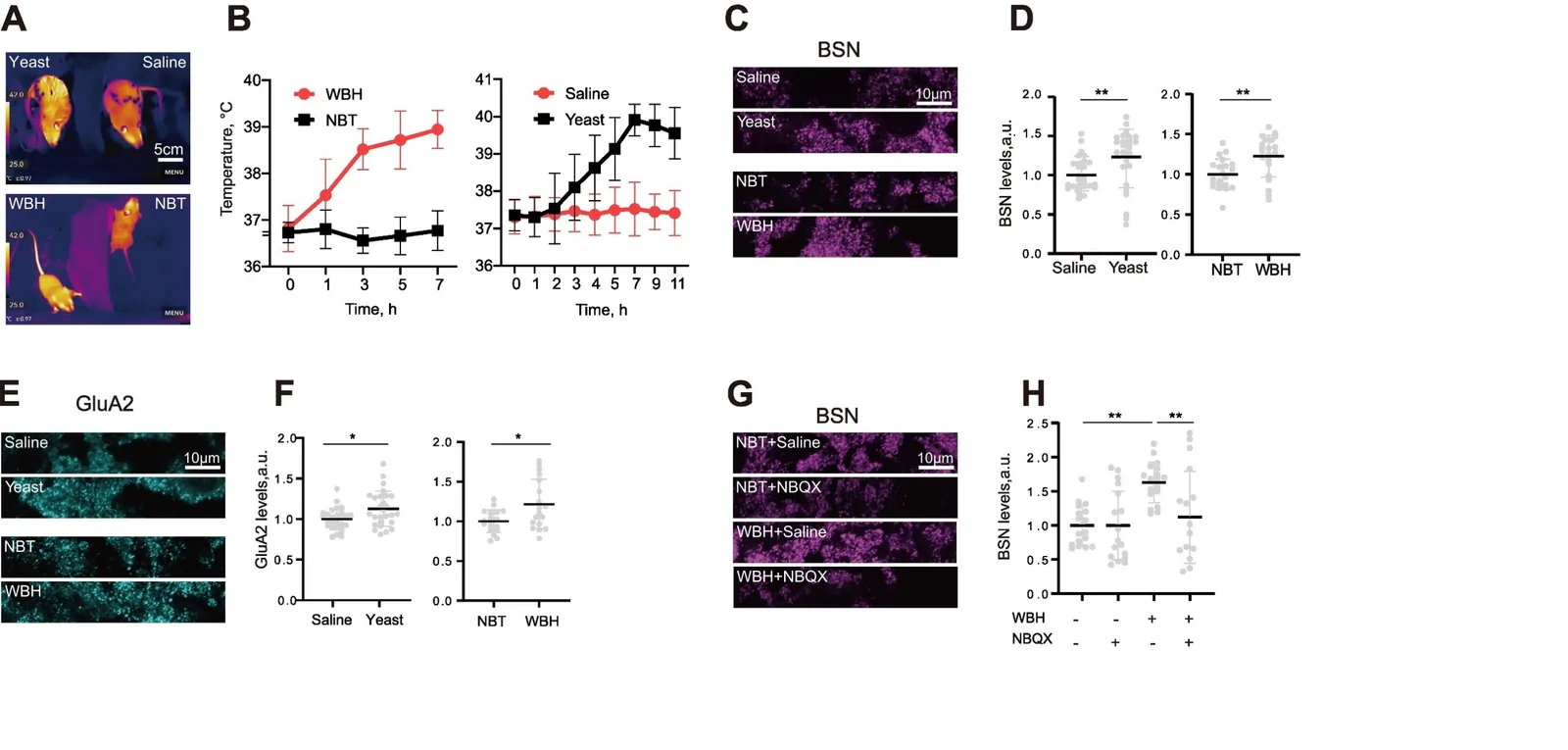
**Fever induces synaptic strengthening through AMPARs**. (**A**) Pseudo-colored thermal images show temperature increase in rats and mouse models. Top, rats, bottom, mice. Left, treated animal, right, control animal. (**B**) Time course of temperature increases in pyrexia and WBH models and saline controls following protocol induction at t = 0. (**C**) Images of brain sections from control and treated animals showing immunostaining for BSN. (**D**) Quantification of temperature effect on BSN. **P < 0.01, Student t test; **P < 0.01, Mann-Whitney test. (**E**) Images of brain sections from control and treated animals with immunostaining for GluA2. (**F**) Quantification of temperature effect on synaptic GluA2 levels. *P < 0.05, Student t-test. (**G**) Images of brain sections showing the effect of NBQX injection on hyperthermia-induced increase in BSN staining. (**H**) Quantification of the NBQX effect on BSN. **P < 0.01, Kruskal-Wallis test with Dunn’s multiple comparisons test. N=9/group, pyrexia; 7/group, WBH; 6/group, NBQX+WBH.

Our previously published evidence demonstrated that temperature decrease induces synaptic remodeling [7]. To investigate synaptic effects of temperature elevation within the physiological range *in vivo*, we leveraged two well-established rodent models of fever, namely pyrexia by yeast injection in rats [8, 9] and WBH in mice [10], and visualized synapses in the prefrontal cortex (PFC), using immunohistochemistry staining for synaptic marker proteins in brain sections to quantify synaptic structure and composition (Supplementary Fig. 1A). Both approaches reliably increased body temperature by 2-3°C within 7 h of treatment (Fig. 1A, B). Intensity of punctate staining for a presynaptic terminal protein Bassoon (BSN) was increased in both models (Fig. 1C, D), as was the case for an excitatory postsynaptic density marker Homer (Supplementary Fig. 1B, C). Staining intensity for several other synaptic markers was also increased, including Ca_V_2.1, vGlut1, gephyrin, GABRA1, synapsin 1/2, and synaptotagmin 1 (Supplementary Fig. 1D-L, 2E-H), broadly confirming the synaptic enhancement in response to temperature increase.

Given the key role of glutamatergic neurotransmission in synaptic plasticity, we investigated glutamate receptors, finding an increase in staining for AMPA receptor GluA2 (Fig. 1E-F), but not NMDA receptor GluN1 (Supplementary Fig. 2A, B). Consistent with a role for AMPARs, blockade of AMPARs by a specific antagonist NBQX blocked temperature-induced increase in staining for BSN (Fig. 1G, H), Homer (Supplementary Fig. 2C, D), Synapsin 1/2 (Supplementary Fig. 2E, F) and Synaptotagmin 1 (Supplementary Fig. 2G, H). Heat shock proteins Hsp70 and Hsc70 were up-regulated by temperature elevation (Supplementary Fig. 2I-K), while intraperitoneal injections of either translation inhibitor anisomycin and Hsp70 inhibitor apoptozole inhibited synaptic enhancement in Homer and BSN (Supplementary Fig. 2L-O). Thus, AMPAR signaling and heat shock translational response are required for temperature-dependent synaptic enhancement.

**Figure 2.**
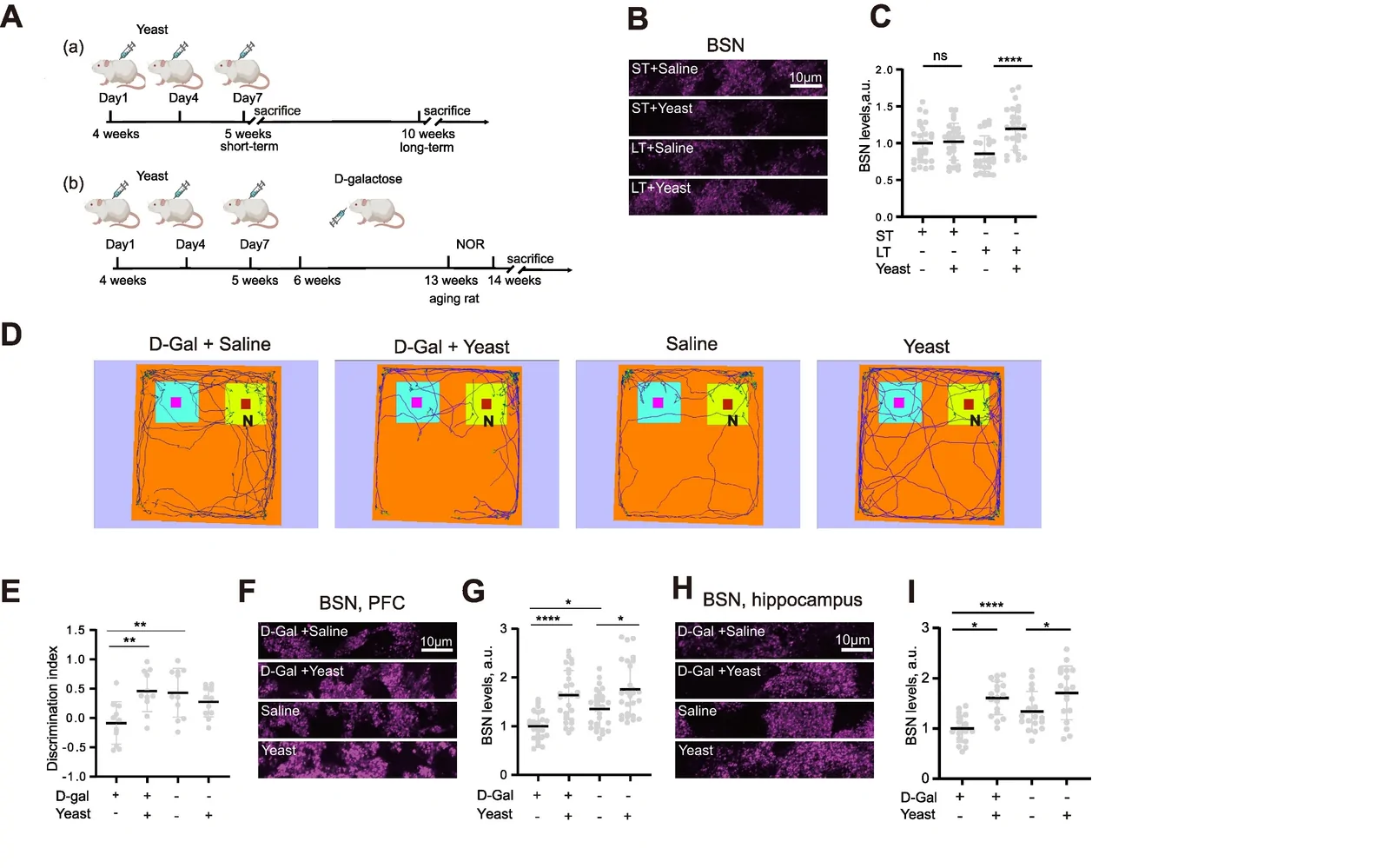
**Juvenile fever induces long-term synaptic strengthening and preserves synapses in a model of aging**. (**A**) Schematics of the experiments for study of the short- and long-term effects (ST and LT) of juvenile pyrexia in rats. (a) - effects of juvenile fever on synapses; (b) - effects of juvenile pyrexia on learning in the model of accelerated aging induced by D-Gal. (**B**) Images of brain sections showing BSN staining after injection of saline or yeast at 4 weeks of age (ST) or 6 weeks later at 10 weeks (LT). (**C**) Quantification of BSN after injection of saline or yeast at 4 weeks (ST) or 6 weeks later at 10 weeks (LT). (**D**) Representative trajectories during the NOR task. Old object, left (azure); Novel object, right (yellow). D-Gal + saline, saline injection at 4 weeks followed by D-Gal treatment; D-Gal + yeast, yeast injection at 4 weeks followed by D-Gal treatment; saline, saline injection at 4 weeks; yeast, yeast injection at 4 weeks. (**E**) Quantification of discrimination indices in control or D-Gal-treated rats. **P < 0.01, 1-way ANOVA with multiple comparisons test. (**F**) Images of brain sections showing BSN staining in PFC of control or D-Gal-treated rats. (**G**) Quantification of BSN in PFC of control or D-Gal-treated rats. ****P < 0.0001, *P < 0.05, Kruskal-Wallis test with Dunn’s multiple comparisons test. (**H**) Images of brain sections showing BSN staining in the hippocampal CA1 region from control or D-Gal-treated rats. (**I**) Quantification of BSN in the hippocampal CA1 region in control or D-Gal-treated rats. ****P < 0.0001, *P < 0.05, 1-way ANOVA with multiple comparisons test. N=9/group, ST and LT pyrexia; 11/group, NOR test; 9/group, PFC staining; 6/group, hippocampus staining.

Previous evidence linking febrile seizures with persistent alterations in neuronal excitability remains inconsistent [1, 2, 5, 6, 11], indicating that temperature and seizures may have distinct effects on juvenile brain connectivity [2]. To investigate the long-term effects of early-age temperature increase on synapses, we induced pyrexia by yeast injection in rats at 4 weeks of age [9], followed by immunohistochemistry at 4 and 10 weeks of age (Fig. 2Aa, Supplementary Fig. 3A, B). Pyrexia had no effect on BSN at 4 weeks, indicating the lack of immediate synaptic strengthening in juvenile animals; there were however significantly more BSN puncta in 10-week old animals subjected to pyrexia at 4 weeks than in the age-matched controls, indicating long-term synaptic enhancement (Fig. 2B, C); similar effect was observed for Homer and GluA2 (Supplementary Fig. 3C-F), but not for other synaptic markers (Supplementary Fig. 3G-N). Levels of Hsp70 and Hsc70 in adult rats were increased at both 4 and 10 weeks, indicating long-term persistence of heat shock response (Supplementary Fig. 3O-R).

To test whether juvenile febrile experience can protect against later synaptic dysregulation, we leveraged the D-galactose (D-Gal) model of aging (Fig. 2Ab), which manifests accelerated synaptic loss and memory decline [12]. We then used the novel object recognition test (NOR) to assess the combined effects of D-Gal and juvenile pyrexia experience on learning and synaptic structure. At 13 weeks, total observation time and discrimination index were significantly lower in D-Gal-treated rats than in controls (Fig. 2D, E, Supplementary Fig. 4A). This cognitive defect was mirrored by a decrease in BSN and Homer intensity as measured by immunostaining in the PFC and the hippocampus at 14 weeks, indicative of synaptic loss accompanying learning deficits (Fig. 2F-I, Supplementary Fig. 4D-G). Conversely, animals with a prior experience of pyrexia at 4 weeks of age showed no D-Gal-induced decline in NOR and synaptic staining, suggesting that juvenile fever conferred a protective effect on synapses and preserved learning ability (Fig. 2D-I, Supplementary Fig. 4A, C, D-G). There were no significant differences in total travelled distances between experimental groups (Supplementary Fig. 4B), indicating that overall mobility was not affected by treatments. Thus, prior experience of juvenile pyrexia protected cognitive function against the D-Gal-induced decline.

## DISCUSSION

Our study presents evidence that moderate fever triggers long-term enhancement of PFC synapses *in vivo*, preserving cognition in a model of aging. On a systemic level, AMPAR- and translation-dependent synaptic enhancement evokes parallels to homeostatic scaling [13], although other temperature-dependent processes may be of relevance, *e.g.* spreading cortical depression associated with febrile seizures [14]. Candidate cellular and molecular mechanisms linking temperature increase with synaptic strengthening include cytoskeletal dynamics [15], neurotrophin release [16, 17], and membrane trafficking modulation via heat shock response [18, 19]; a recently demonstrated direct enhancement of AMPAR activity via temperature-dependent glutamate gating may also play a role [20]. Mechanistic understanding of fever-induced synaptic remodelling warrants further in-depth investigation.

At this moment, the physiological role of fever-induced synaptic strengthening can be but speculated upon. To begin with, it will be important to determine whether the protective effect of fever on brain functionality represents an evolutionarily conserved feature akin to the key role of fever in immune response [21] or an unintended consequence of temperature increase. While it is tantalising to suggest synaptic function as a downstream effector of brain temperature variability and variation [22], there is currently no evidence to support this notion. The above topics can be addressed through a detailed appraisal of the relationship between temperature and synaptic plasticity in a variety of experimental systems, ultimately involving PET imaging of synapses in the living human brain [23].

Further work will be necessary to establish the clinical relevance of our study’s findings, given its key experimental limitations, in particular use of males to avoid the confounding cause-effect relationship between temperature and the estrous cycle [24, 25], as well as use of an accelerated aging model. Evidence linking hyperthermia and brain pathology has been generally inconclusive, reporting both positive [26-28] and negative [29, 30] outcomes, highlighting the complex relationship between temperature and brain function. Further exploration of the putative therapeutic potential of fever for synaptic protection will necessitate systemic investigation of temperature-dependent synaptic remodelling in the context of natural aging in both sexes, with a particular focus on age-related synaptopathies.

### Study approval

All animal use and experimental protocols were approved and carried out in compliance with the IACUC guidelines and the Animal Care and Ethics Committee of Qingdao University School of Medicine (approval number QDU-HEC-2024458).

## Supplementary Materials

The Supplementary data can be found online at: www.aginganddisease.org/EN/10.14336/AD.2025.0591.



## Data Availability

Raw experimental data is available from the Authors upon written request.
