# Supplementary material for: Fever Induces Long-Term Synaptic Enhancement and Protects Learning in an Accelerated Aging Model
Source: Aging Dis. 2026 Jul 21;17(5):2577–82. doi: 10.14336/AD.2025.0591 (PMC13437104; doi:10.14336/AD.2025.0591)
Supplement: Supplementary file 1 [file AD-17-5-2577-s.pdf]

SUPPLEMENTARY DATA

# Fever Induces Long-Term Synaptic Enhancement and Protects Learning in an Accelerated Aging Model

**Fusheng Du, Qi Wan, Oleg O. Glebov**

© 2025. Du F et al. Published online at http://www.aginganddisease.org/EN/10.14336/AD.2025.0591

# SUPPLEMENTARY DATA

## EXTENDED METHODS

### Key materials

Experimental animals were from Jinan Pengyue Laboratory Animal Co. Apoptozole and NBQX were from AbMole. Anisomycin and D-galactose were from Macklin. Yeast was from Angle Yeast. Antibodies used in this study are detailed in **Supplementary Table 1**.

### Animals

Male, 8-10 week-old, 28-32 g of weight, specific pathogen free (SPF), Institute of Cancer Research (ICR) mice were used in the whole-body hyperthermia model. Male, 8-10 week-old, 230-250 g of weight, SPF Sprague Dawley (SD) rats were used in the yeast-induced pyrexia model. Male, 3-4 week-old, 60-80 g of weight, SPF SD rats were used in the juvenile pyrexia model.

All the animals were housed in the animal facility of Qingdao University, under conditions adhering to the GB 14925-2010 standard of Laboratory Animal Environment and Facilities. Specifically, the temperature was maintained between 18-26℃, with a day-night temperature difference not exceeding 4℃. The relative humidity was kept at 40-70% to prevent proliferation of pathogens. The ventilation system provided 10-20 air exchanges per hour, with ammonia concentrations not exceeding 14 mg/m³. The lighting cycle was set at 12 hours of light followed by 12 hours of darkness, with a lighting intensity of 150-300 lx. Noise levels were controlled to maintain the animals' normal physiological rhythms and reduce stress responses. Animal rooms were maintained at the SPF-grade cleanliness standard.

### Sex as a biological variable

Male animals were used throughout the study.

### Injection of inhibitors

NBQX, anisomycin and apoptozole were injected intraperitoneally one hour before the whole body hyperthermia model. The control group was given the same amount of normal saline at the concentrations of 10 mg/kg, 150 mg/kg and 4 mg/kg respectively. The intraperitoneal injection volume was 100 μl per animal.

### Immunohistochemistry staining

After treatment, animals were anesthetized with isoflurane and then perfused transcardially with normal saline. The brains were dissected out and kept at -80℃. Coronal sections (25 um) were cut at -20℃ with a cryostat (Leica CM1950). Sections were made at approximately +1.5 to +3, +1.5 to +3 and +2 to +4 mm from bregma in mice, juvenile rats and adult rats respectively. then fixed with 4% PFA for 20 mins at RT and washed three times for 10 mins each in PBS. Sections were incubated in blocking buffer (10% goat serum, 0.3% Triton-X 100 in PBS) for 1 hour at RT, and incubated with primary antibodies for 36-48h at 4℃, followed by 1 hour at RT. Sections were washed six times for 5 mins each in PBS, followed by incubating with Alexa Fluor 488- and Alexa Fluor 594-conjugated secondary antibodies for 75 mins at RT, and washed three times for 10 mins each in PBS. Then all sections were mounted onto positively-charged microscope slides and covered in DAPI-containing mount media (Solarbio). To control for

© 2025. Du F et al. Published online at http://www.aginganddisease.org/EN/10.14336/AD.2025.0591

specificity of staining, negative controls were conducted with only secondary antibodies, showing that the signal intensity due to non-specific binding was <5%.

**Confocal microscopy**

Samples were imaged on Nikon Eclipse Ti2 laser confocal microscope. The imaging systems were controlled by NIS Elements 2.0 software. The following image acquisition settings were used for serial confocal z-stack images: tissue section imaging of synaptic proteins, 0.5 μm step, 2 μm range, 512 x 512 pixels image size, 2x zoom, 100x magnification; tissue section imaging of Hsp70 and Hsc70, 512 x 512 pixels image size, 1x zoom, 20x magnification. Pinhole size was kept between 1-2 Airy units. Excitation laser wavelengths were 488 nm and 561 nm. Bandpass filters were set at 500−550 nm (FITC, Alexa Fluor 488) and 570−620 nm (TRITC, AlexaFluor 594). Gain, exposure and offset settings were optimized within each experiment to ensure appropriate dynamic range, low background and optimal signal/noise ratio.

**Analysis of synaptic structure and composition**

The schematics of image analysis are presented in **Supplementary Fig. 1A**. To identify individual synapses, images were binarized in ImageJ using the "Moments" setting, and particles were counted automatically using the "Analyze Particles" command across the whole image. To avoid instances of multiple synapses overlapping, only ROIs with areas ranging from 0.1 to 2 $\mu m^2$ were included in further analysis. All values of circularity were included in analysis. Individual ROIs were then combined into one compound ROI using the "Combine" and "Add" functions of the ROI Manager interface, whereupon quantification of mean signal intensity in each channel was performed using the "Measure" function. Since background fluorescence intensity was typically less than 1% of the median fluorescence in each channel within a ROI, background subtraction did not significantly affect the measurements and was not performed.

**Statistical analysis**

All the experiments were performed in at least 6 independent biological replicates, with three fields of view from one brain section per animal used for analysis. Statistical analysis was carried out using the GraphPad Prism v5 (https://www.graphpad.com/). For each experiment, data was normalized to the average value in the control sample. Data distributions were assessed for normality using d'Agostino and Pearson omnibus normality tests. For normally distributed datasets, two sample t test and 1-way ANOVA, with Dunn's, Bonferroni's post tests were used to assess statistical significance as appropriate; for non-normally distributed datasets, Mann-Whitney rank test, Kruskal-Wallis test and Dunn's post test were used. The data sets are presented in the form of scatter plots and line plots, with plot averages as lines.

© 2025. Du F et al. Published online at http://www.aginganddisease.org/EN/10.14336/AD.2025.0591

# SUPPLEMENTARY DATA

## SUPPLEMENTARY FIGURES

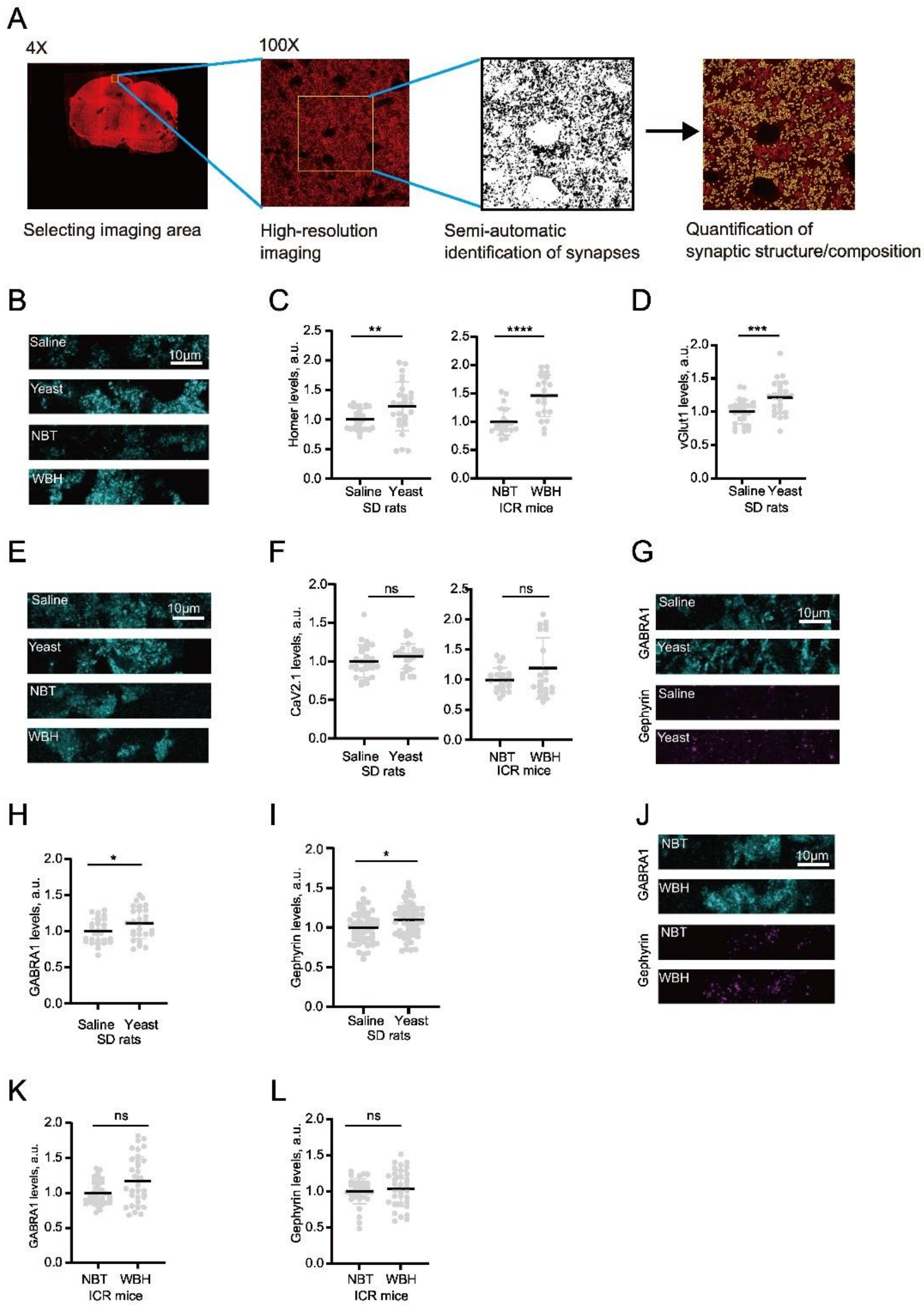


**Supplementary Figure 1. Fever effects various synaptic markers. (A)** Schematic of the experimental approach to quantification of synaptic structure and composition. For details, see Materials and Methods, sections "Confocal Microscopy" and "Image Analysis". NB: The images provided are for illustration of the methodology, *i.e.* the selected area does not necessarily represent the brain region used in measurements. **(B)** Images of brain sections from control and treated animals with immunostaining for Homer. **(C)** Quantification of temperature effect on Homer levels in control and treated animals. **(D)** Quantification of temperature effect on vGlut1 levels in control and treated animals. **(C)** Images of brain sections from control and treated animals with immunostaining for Cav2.1. **(F)** Quantification of temperature effect on Cav2.1 levels in control and treated animals. **(G)** Images of brain sections from control and yeast-injected animals with immunostaining for

© 2025. Du F et al. Published online at http://www.aginganddisease.org/EN/10.14336/AD.2025.0591

GABRA1 and gephyrin. **(H)** Quantification of temperature effect on GABRA1 levels in control and yeast-injected animals. **(I)** Quantification of temperature effect on gephyrin levels control and yeast-injected animals. **(J)** Images of brain sections from control and WBH-treated animals with immunostaining for GABRA1 and gephyrin. **(K)** Quantification of temperature effect on GABRA1 levels in control and WBH-treated animals. **(L)** Quantification of temperature effect on gephyrin levels control and WBH-treated animals. ****P<0.0001, ***P<0.001, **P<0.01, *P<0.05, ns - not significant. N=9/group, pyrexia; 7/group, WBH.

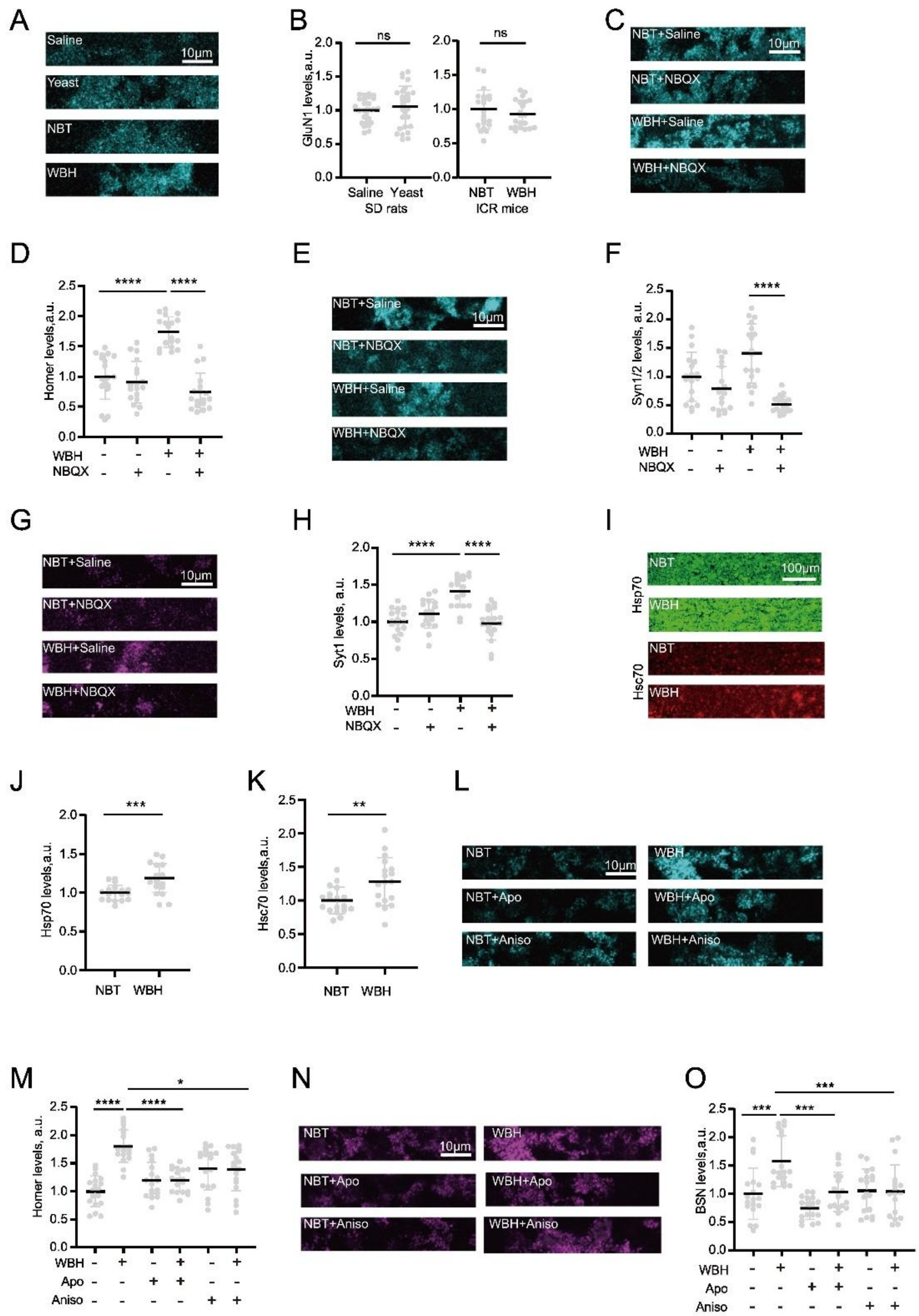


**Supplementary Figure 2. Mechanisms of synaptic enhancement by fever. (A)** Images of brain sections from control and treated animals with immunostaining for GluN1. **(B)** Quantification of temperature effect on GluN1 levels in control and

treated animals. **(C)** Images of brain sections from control and WBH-treated animals showing NBQX effect on WBH-induced increase in Homer. **(D)** Quantification of NBQX effect on WBH-induced increase in Homer. **(E)** Images of brain sections from control and WBH-treated animals showing WBH-induced increase in synapsin 1/2 and NBQX effect thereon. **(F)** Quantification of WBH-induced increase in synapsin 1/2 and NBQX effect thereon. **(G)** Images of brain sections from control and WBH-treated animals showing WBH--induced increase in synaptotagmin 1 and NBQX effect thereon. **(H)** Quantification of WBH-induced increase in synaptotagmin 1 and NBQX effect thereon. **(I)** Images of brain sections from control and WBH-treated animals showing WBH-induced increase in Hsp70 and Hsc70. **(J)** Quantification of WBH-induced increase in Hsp70. **(K)** Quantification of WBH-induced increase in Hsc70. **(L)** Images of brain section from control and treated animals showing effects of apoptozole and anisomycin application on temperature-induced Homer levels. **(M)** Quantification of the effects of apoptozole and anisomycin application on temperature-induced Homer levels. **(N)** Images of brain section from control and treated animals showing effects of apoptozole and anisomycin application on BSN staining. **(O)** Quantification of the effects of apoptozole and anisomycin application on BSN. ****P<0.0001, ***P<0.001, **P<0.01, *P<0.05, ns - not significant. N≥6 independent biological replicates. N=9/group, pyrexia; 7/group, WBH; 6/group, NBQX+WBH; 6/group, Anisomycin+apoptozole.

© 2025. Du F et al. Published online at http://www.aginganddisease.org/EN/10.14336/AD.2025.0591

# SUPPLEMENTARY DATA

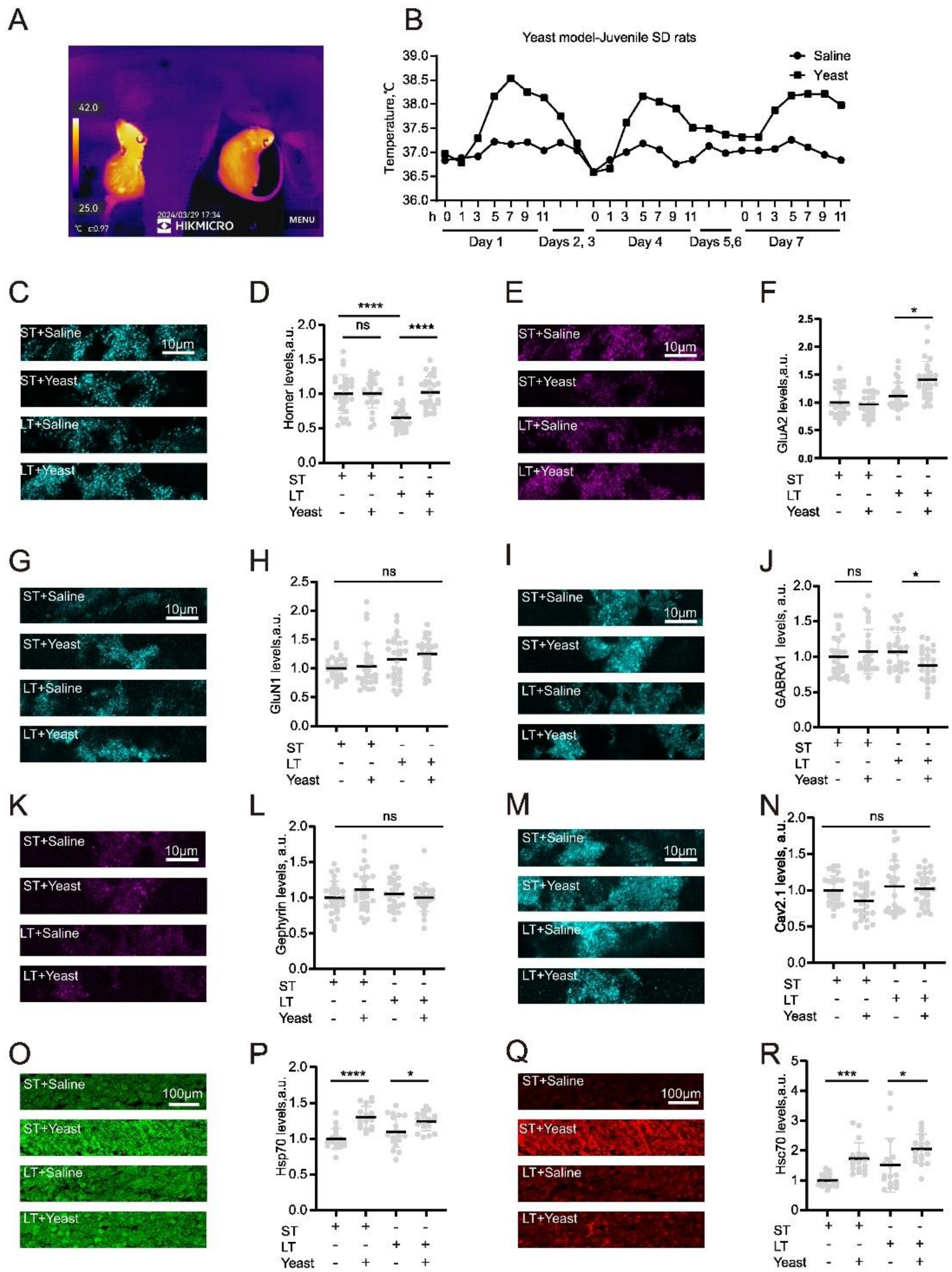


**Supplementary Figure 3. Short- and long-term synaptic changes following juvenile pyrexia. (A)** Images of a 4-week old rat subjected to pyrexia induction by yeast injection (left) and a control rat with saline injection (right). **(B)** Example temperature traces showing repeated bouts of temperature elevation during juvenile pyrexia. NB: The x axis is non-linear. **(C)** Images of brain sections showing the effect of juvenile pyrexia on Homer levels at 4 (short-term, ST) and 10 (long-term, LT) weeks. **(D)** Quantification of the juvenile pyrexia effect on Homer levels at 4 (short-term, ST) and 10 (LT) weeks. N=9. **(E)** Images of brain sections showing the effect of juvenile pyrexia on GluA2 levels at 4 (short-term, ST) and 10 (long-term, LT) weeks. **(F)** Quantification of the juvenile pyrexia effect on GluA2 levels at 4 (short-term, ST) and 10 (long-term, LT) weeks. **(G)** Images of brain sections showing the effect of juvenile pyrexia on GluN1 levels at 4 (short-term, ST) and 10 (long-term, LT) weeks. **(H)** Quantification of the juvenile fever effect on GluN1 levels at 4 (short-term, ST) and 10 (LT)

weeks. **(I)** Images of brain sections showing the effect of juvenile pyrexia on GABRA1 levels at 4 (short-term, ST) and 10 (long-term, LT) weeks. **(J)** Quantification of the juvenile pyrexia effect on GABRA1 levels at 4 (short-term, ST) and 10 (long-term, LT) weeks. **(K)** Images of brain sections showing the effect of juvenile pyrexia on gephyrin levels at 4 (short-term, ST) and 10 (long-term, LT) weeks. **(L)** Quantification of the juvenile pyrexia effect on gephyrin levels at 4 (short-term, ST) and 10 (long-term, LT) weeks. **(M)** Images of brain sections showing the effect of juvenile pyrexia on CaV2.1 levels at 4 (short-term, ST) and 10 (long-term, LT) weeks. **(N)** Quantification of the juvenile pyrexia effect on CaV2.1 levels at 4 (short-term, ST) and 10 (long-term, LT) weeks. **(O)** Images of brain sections showing the effect of juvenile pyrexia on Hsp70 levels at 4 (short-term, ST) and 10 (long-term, LT) weeks. **(P)** Quantification of the juvenile pyrexia effect on Hsp70 levels at 4 (short-term, ST) and 10 (long-term, LT) weeks. **(Q)** Images of brain sections showing the effect of juvenile pyrexia on Hsc70 staining at ST and LT. Images represent the same areas as in panel O. **(R)** Quantification of the juvenile pyrexia effect on Hsc70 levels at 4 (short-term, ST) and 10 (long-term, LT) weeks. ns-not significant, *P < 0.05, ***P < 0.001,****P < 0.0001, Kruskal-Wallis test with Dunn's multiple comparisons test. N=9/group, ST and LT pyrexia.

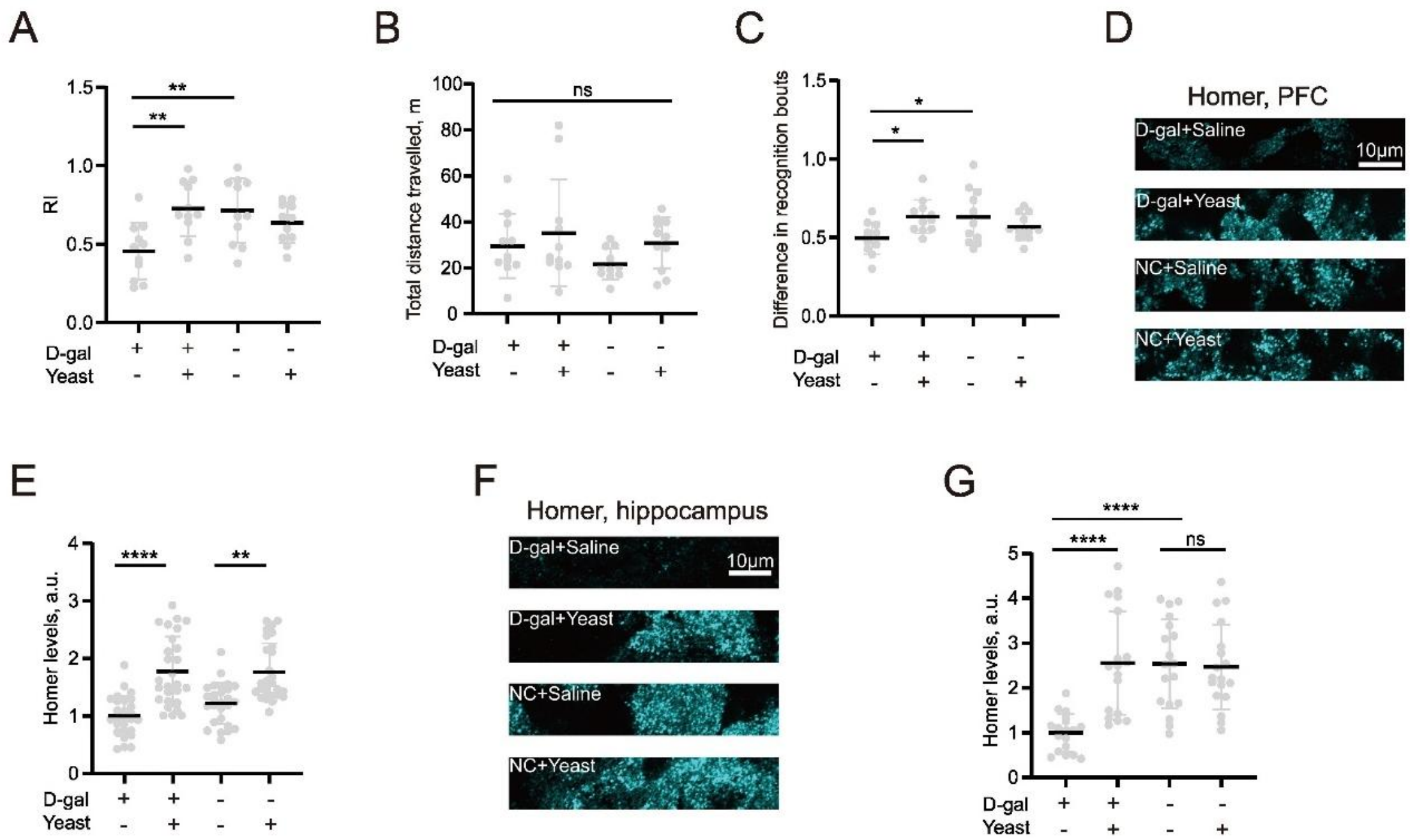


**Supplementary Figure 4. Long-term effects of juvenile pyrexia in the D-Gal injected rats. (A)** Effects of juvenile pyrexia on recognition index in control and D-Gal-injected rats with or without prior juvenile febrile experience. **(B)** Effects of juvenile pyrexia on total distance in control and D-Gal-injected rats with or without prior juvenile febrile experience. **(C)** Effects of juvenile pyrexia on Recognition Bouts in control and D-Gal-injected rats with or without prior juvenile febrile experience. **(D)** Images of brain sections showing Homer staining in PFC of control or D-Gal-injected rats with or without prior juvenile pyrexia. Images represent the same areas as in Figure 2F. **(E)** Quantification of Homer levels from the PFC in control or D-Gal-injected rats with or without prior juvenile pyrexia. ns-not significant, *P < 0.05, **P < 0.01,****P < 0.0001, Kruskal-Wallis test with Dunn's multiple comparisons test. **(F)** Images of brain sections showing Homer staining in the hippocampal CA1 region from control or D-Gal-injected rats with or without prior juvenile pyrexia. Images represent the same areas as in Figure 2H. **(G)** Quantification of Homer levels from the hippocampal CA1 region (HC-CA1) in control or D-Gal-injected rats with or without prior juvenile pyrexia. ****P < 0.0001, ns-not significant, 1-way ANOVA with multiple comparisons test. N=11/group, NOR test; 9/group, PFC staining; 6/group, HC staining.

© 2025. Du F et al. Published online at http://www.aginganddisease.org/EN/10.14336/AD.2025.0591

# SUPPLEMENTARY DATA

**Supplementary Table 1. Antibodies used in this study.**

| Antigen | Conjugation | Species | Manufacturer | Cat. No. | Dilution |
|---|---|---|---|---|---|
| Gephyrin | - | mouse | Synaptic Systems | 147011 | 1/500 |
| vGlut1 | - | Rabbit | Synaptic Systems | 135302 | 1/1000 |
| Synapsin1/2 | - | Rabbit | Synaptic Systems | 106002 | 1/1000 |
| GABRA1 | - | Rabbit | Synaptic Systems | 224203 | 1/1000 |
| BSN | - | mouse | Abcam | ab82958 | 1/1000 |
| Cav2.1 | - | Rabbit | Synaptic Systems | 152203 | 1/1000 |
| Synaptotagmin1 | - | mouse | Synaptic Systems | 105311 | 1/1000 |
| GluA2 | - | mouse | Merck Millipore | 3230950 | 1/1000 |
| Homer | - | Rabbit | Synaptic Systems | 160002 | 1/1000 |
| GluN1 | - | Rabbit | Alomone Labs | AGC001an0702 | 1/500 |
| Rabbit IgG | AlexaFluor488 | goat | Bioss | bs-0295G-AF488 | 1/500 |
| Mouse IgG | AlexaFluor594 | goat | SolarBio | K1031G-AF594 | 1/500 |
| Anti-HSP70 Antibody | - | rabbit | Boster | BA0928 | 1/400 |
| Anti-HSC70/HSPA8 Antibody | - | mouse | Boster | M01024-1 | 1/400 |

© 2025. Du F et al. Published online at http://www.aginganddisease.org/EN/10.14336/AD.2025.0591
